# 

**Published:** 1914-10-15

**Authors:** 


### ANNOUNCEMENTS.

FEDERATION DENTAIRE INTERNATIONALE. At the annual meeting of the International Dental Federation, London, England, August 6, 1914, the following officers were elected for 1914-15.

*Hon. President*—W. B. Paterson, London.

*President*—Truman W. Brophy, Chicago.

*Vice-Presidents*—Harvey J. Burkhart, Batavia, N. Y.; F. Schaeffer, Stuckert, Frankfort-on-Main; M. Roy, Paris; W. Guy, Edinburg; Rudolph Weiser, Vienna; Vincenzo Guerini, Naples; J. Howard Mummery, London; N. Etchepareborda, Buenos Ayres; Ernst Jessen, Strassburg.

*Secretary-General*—Florestan Aguilar, Madrid.

*Assistant Secretaries*—Burton Lee Thorpe, St. Louis; C. Van der Hoeven, The Hague; G. Villain, Paris; B. Landete, Madrid.

*Treasurer*—Edmond Rosenthal, Brussels.

Next place of meeting San Francisco, August 30, 1915.

BURTON LEE THORPE,
*Asst. Secy.*

OHIO STATE DENTAL SOCIETY.—The Forty-Ninth Annual Meeting of the Ohio State Dental Society will convene in Memorial Hall, Columbus, Tuesday, December 1, continuing through the 2d and 3d. Papers on live subjects by able men on Tuesday and Wednesday afternoon; clinics, Wednesday and Thursday forenoons.

Papers will be presented by Drs. W. A. Griffin, of Detroit; H. W. McMillan, Cincinnati; W. W. Curtiss, Greenfield; and J. R. Callahan, Cincinnati.

The program is not sufficiently perfected to announce at this time, but a strong program and big meetings are assured.

The entire state is now organized into components and a very large attendance is anticipated.

F. R. CHAPMAN, *Sec'y.*

MICHIGAN BOARD MEETING.—The semi-annual meeting of The Michigan State Board of Dental Examiners will be held in the Dental College at Ann Arbor, commencing Monday, November 9, and continuing through the 14th. For full particulars and application blanks address,

F. E. SHARP, *Secretary.*
Port Huron, Mich.

MICHIGAN LICENTIATES PLEASE NOTE.—The licenses of all Michigan licentiates, whether practicing in the State or not, who have not paid their annual registration fee, will be revoked at the next regular meeting of the Board, which will be held in Ann Arbor, November 9th to 14th.

F. E. SHARP, *Secretary.*
Port Huron, Mich.

